# Diagnosing and Managing Velopharyngeal Insufficiency in Patients With Cleft Palate After Primary Palatoplasty

**DOI:** 10.1097/SCS.0000000000009822

**Published:** 2023-11-13

**Authors:** Veera V. Pitkanen, Ahmed Geneid, Anne M. Saarikko, Sanna Hakli, Suvi A. Alaluusua

**Affiliations:** * Cleft and Craniofacial Center, Department of Plastic Surgery, Helsinki University Hospital and University of Helsinki; † Department of Otolaryngology and Phoniatrics—Head and Neck Surgery, Helsinki University Hospital and University of Helsinki, Helsinki; ‡ Department of Otolaryngology and Phoniatrics, Oulu University Hospital and PEDEGO Research Unit and Medical Research Center Oulu, University of Oulu, Oulu, Finland

**Keywords:** Cleft palate, velopharyngeal insufficiency, VPI, velopharyngeal dysfunction

## Abstract

Velopharyngeal insufficiency (VPI) after palatoplasty is caused by improper anatomy preventing velopharyngeal closure and manifests as a hypernasal resonance, audible nasal emissions, weak pressure consonants, compensatory articulation, reduced speech loudness, and nostril or facial grimacing. A multidisciplinary team using multimodal instruments (speech analysis, nasoendoscopy, videofluoroscopy, nasometry, and magnetic resonance imaging) to evaluate velopharyngeal function should manage these patients. Careful monitoring of velopharyngeal function by a speech pathologist remains paramount for early identification of VPI and the perceptual assessment should follow a standardized protocol. The greatest methodology problem in CLP studies has been the use of highly variable speech samples making comparison of published results impossible. It is hoped that ongoing international collaborative efforts to standardize procedures for collection and analysis of perceptual data will help this issue. Speech therapy is the mainstay treatment for velopharyngeal mislearning and compensatory articulation, but it cannot improve hypernasality, nasal emissions, or weak pressure consonants, and surgery is the definitive treatment for VPI. Although many surgical methods are available, there is no conclusive data to guide procedure choice. The goal of this review article is to present a review of established diagnostic and management techniques of VPI.

Normal speech with velopharyngeal competence (VPC) is possible with an intact palate separating the oral and nasal cavities. The velopharyngeal sphincter, formed by the soft palate, the lateral pharyngeal walls, and the posterior pharyngeal wall, separates the nasal and oral cavities, controls speech resonance, and prevents regurgitation of food and fluid during swallowing. The levator veli palatini, palatoglossus, palatopharyngeus, musculus uvulae, salpingopharyngeus, and superior pharyngeal constrictors participate in the function of the velopharyngeal sphincter.^1^ This sphincter controls its shape and size, directing the airflow between the nasal and oral cavities, and must be tightly closed to achieve VPC.

Velopharyngeal dysfunction is a condition where the velopharyngeal valve fails to close properly during the production of oral sounds and has multiple causes, including velopharyngeal insufficiency (anatomic defect preventing velopharyngeal closure), velopharyngeal incompetence (neurophysiologic dysfunction causing poor pharyngeal movement), and velopharyngeal mislearning (nasopharyngeal sound substitution for an oral sound)^2^ (Fig. 1). Velopharyngeal dysfunction manifests as a hypernasal resonance, audible nasal emissions, weak pressure consonants, compensatory articulation (including retracted oral articulation, glottal, and nasal or pharyngeal fricatives), reduced speech loudness, and nostril or facial grimacing.^3^

**FIGURE 1:**
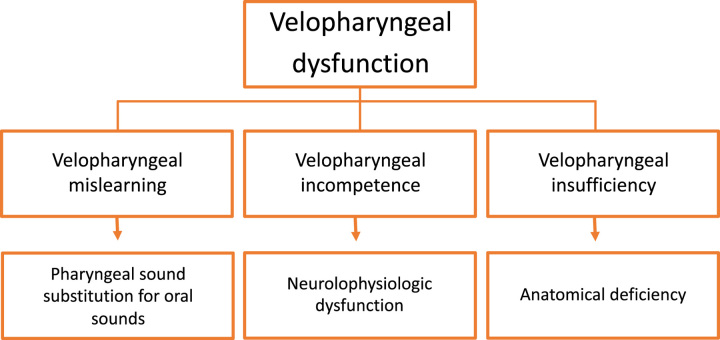
Classification of velopharyngeal dysfunction.

The underlying cause of velopharyngeal dysfunction in cleft patients is usually anatomical, and the condition is called velopharyngeal insufficiency (VPI). Although many causes of velopharyngeal dysfunction exist, this article focuses on VPI in patients with cleft palate. After primary palatoplasty for patients with cleft palate, palatal shortening due to cleft anatomy and surgery, insufficient levator function, and scar contracture usually still cause VPI in cleft patients. Other causes of VPI include submucous cleft palate, velar dysplasia, deep pharynx, irregular adenoids, adenoidectomy, tonsillectomy, maxillary advancement, and oral or pharyngeal cavities.^1^ In addition to anatomical abnormalities, cranial nerve abnormalities and pharyngeal muscle hypotonia may play a role in the development of VPI in cleft patients, especially if a syndrome is involved. Cleft patients may also develop velopharyngeal mislearning. Velopharyngeal insufficiency results in decreased intelligibility of speech, social impairment, and a negative quality of life for patients and their parents.^4^ In addition to VPI, children with clefts are at risk of speech delay and those with persisting speech problems are at risk of impaired acquisition of reading.^5^ Cleft patients with VPI and compensatory articulation are at especially high risk of language development delay, although the reasons are not fully understood.^6^

This review article focuses on diagnosis and management of VPI in cleft palate patients. Diagnostic tools and surgical techniques have evolved in recent years, and more outcome data after surgical and conservative management has been published. Our goal is to present a review of established diagnostic and management techniques.

## DIAGNOSING VPI

Velopharyngeal insufficiency diagnosis and management should be performed by a multidisciplinary team, using both perceptual and instrumental assessment of speech. Instrumental assessment of VPI (Supplemental Table 1, Supplemental Digital Content 1, http://links.lww.com/SCS/F608) aims to more standardized speech outcomes and allows VPI management algorithms. However, careful monitoring of emerging speech and velopharyngeal function by a speech pathologist remains paramount for early identification of VPI, especially when the perceptual assessment follows a standardized protocol. Many modalities can confirm the VPI diagnosis, including: nasometry; nasoendoscopy; videofluoroscopy (multiview and regular); and magnetic resonance imaging (MRI). These modalities are useful for diagnosing VPI. They aid in choosing the surgical method for VPI correction; the visualization of velopharyngeal activity is important for in selecting a treatment method. Videonasoendoscopy and Videofluoroscopy are the primary tools for upper vocal tract examination and both should be videorecorded with sound allowing the multidisciplinary cleft team to review them. The goal of velopharyngeal function investigation is to assess structure, movement, extent closure, and timing. Because the treatment of VPI is variable, investigations should depend on the treatment protocol of a particular team.

### Perceptual Speech Assessment

The primary role of the speech pathologist working in a cleft team is to evaluate weather deviations in oral cavity structures negatively impact speech production.^7^ Detailed methods of perceptual speech assessment are beyond the scope of this article. A speech pathologist first determines whether the speech abnormality is a result of velopharyngeal dysfunction, an abnormality of speech sound learning, or both. Most commonly assessed parameters of velopharyngeal dysfunction are resonance, articulation and nasal emissions using descriptive category judgements for-instance normal, mild, moderate, severe on an interval scaling.^8^

Audio or video recordings are recommended for better reliability and future reference when reporting speech outcomes of individuals with a cleft. In researches on VPI, blind independent analysis of speech data by at least 2 trained speech pathologists with experience of cleft speech evaluation, a narrow age span in subjects, reporting of inclusion and exclusion criteria, and inter- rater and intra-rater reliability should all be incorporated.^8–11^ However, a greater issue in CLP study methodologies include the use of highly variable speech samples and the lack of information about listeners and on reliability, making comparison of published results impossible.^8^ It is hoped that ongoing international collaborative efforts to standardize procedures for collection and analysis of perceptual data will help this issue.

The recommendations of the standardized speech samples can be found on the website “Cleft palate international speech issues” (https://clispi.com/), which was originally developed for the Eurocran project. Four more refined and standardized tools for perceptual evaluation of cleft palate speech have been designed (Supplemental Table 2, Supplemental Digital Content 1, http://links.lww.com/SCS/F608).^12–16^ Standardized procedures have been used in United Kingdom for research, national audits, and education.^17–19^ The Cleft Audit Protocol for Speech-Augmented (CAPS-A) includes evaluation of articulation, resonance, and nasal airflow and has been used in CLP audit studies in the UK and Ireland.^19^ CAPS-A has undergone assessment of face, content and criterion validity, reliability, and acceptability and uses the traffic light system^20^ in scoring cleft speech characteristics (green indicates a satisfactory result with no intervention needed; yellow indicates a need to monitor progress because there may be a requirement for some intervention; red indicates an unsatisfactory result requiring further detailed speech assessment, structural investigations, and probable surgical intervention and/or speech therapy intervention). The Universal Parameters for reporting Speech outcomes in individuals with cleft by Hutters, Henningsson, and colleagues.^21,22^ The recommendations highlighted the importance of phonetically identical sound inventories in the speech material used, including pressure consonants and high vowels for their vulnerability to the cleft condition.^23,24^ Inspired by the Scandcleft project, Lohmander and colleagues made recommendations for standardized speech samples for comparing results between centers and different languages.^25^ More recently, Lohmander and colleagues designed The Swedish Articulation and Nasality Test (SVANTE) inspired by the Scandcleft project; it is used widely in Nordic countries and is also designed to enable crosslinguistic evaluation.^26^ Ongoing recent international randomized study TOPS (Timing Of Primary Surgery for Cleft Palate) trial uses speech assessment methodology developed within the Scandcleft study with the addition to include Brazilian Portuguese.^27^ Differences between the 4 standardized perceptual assessment protocols are presented in Supplemental Table 3, Supplemental Digital Content 1, http://links.lww.com/SCS/F608.

### Rating of Velopharyngeal Function

As with perceptual evaluation, there is a significant variability in the literature regarding the outcomes of surgery for velopharyngeal function.^28^ Overall ratings of velopharyngeal function are associated with speech symptoms related to inadequate velopharyngeal function and have been used as a general perceptual estimate of velopharyngeal function, and similarly a sum of ratings of speech symptoms related to VPI has been shown to be reliable.^29^ However, in de Blacam and colleagues recent systematic review, only 5/83 studies used a published, validated scale for reporting perceptual speech outcome.^30^

A composite variable (VPC-Sum) was created for the Scandcleft project and first version of it was validated within the Scandcleft methodology^25^ and later updated and it’s validation verified.^29^ VPC-Sum is a summary on scores 0 to 2 (0 competent, 1 marginally incompetent, 2 incompetent) on hypernasality, active, non-oral errors, and symptoms of VPI (audible nasal air leakage, weak pressure consonants, and nasal realizations of consonants) and the sum is presented similarly with a 3-point scale: 0 competent (0,1); 1 marginally incompetent (2,3); and incompetent (4-6).^6^ Overall auditory perceptual estimation of the velopharyngeal closure (VPC-Rate) was also originally preliminarily validated within the Scandcleft study^25^ and later updated and validated.^29^ VPC-rate is scored similar to VPC-Sum through a 3-point scale and based on connected speech; its major advantage is that it is quick to use and valuable in the clinical setting compared with VPC-Sum, the calculation of which is more time-consuming and recommended for in-depth research studies.^29^ The SVANTE protocol used in Scandinavia uses categorical rating of velopharyngeal function and also VPC-Rate.^26^ CAPS-A did not originally include a common rate of velopharyngeal function in the methodology^19^; later however, Pereira and colleagues validated VPC-Sum CAPS-A using CAPS-A methodology within their osteotomy study.^31^

Another issue is that the definition of success of VPI surgery is absence of consistency and definition of normal or acceptable speech intelligibility. Most studies use subjective descriptors and the most commonly used criteria for success after palatoplasty is “acceptable speech”, which is hard to define and always subjectively to the evaluator; some studies include patients with borderline or mild VPI in their success category, whereas others include only patients with completely normal VPC.^28^ Both standardized perceptual evaluation and standardized rating of velopharyngeal function are mandatory to compare postoperative results of patients with CLP.

### Nasometry

**FIGURE 2:**
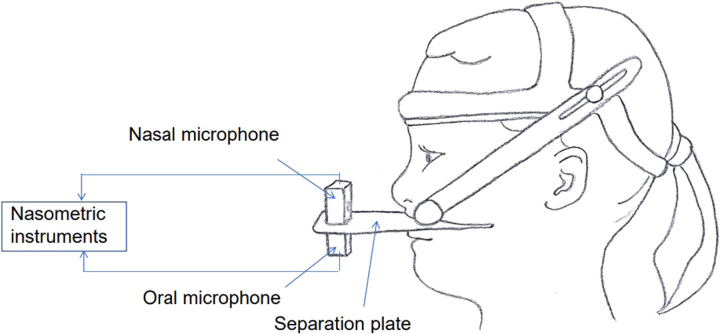
Nasometer consists of a headset with 2 microphones, positioned in front of the nose and mouth, connected to nasometric instruments and a separation plate. Picture illustrated by Satu Alaluusua.

Nasometry is a computer-based procedure used to measure the acoustic correlates of resonance and air escaping past the velum and through the nose, providing indirect information regarding the function of the velopharyngeal sphincter.^32^ A nasometer consists of a headset with 2 microphones, positioned in front of the nose and mouth, connected to nasometric instruments and a separation plate (Fig. 2). Nasometry provides important instrumental and objective evaluation of nasality, which can be difficult for even an experienced cleft speech pathologist.^33^ During speech, nasometry records data of acoustic energy from nasal and oral cavities, and calculates the average ratio of nasal and total (nasal and oral) acoustic energy in a percentage value for the nasalance score.^32^ These scores are compared with normative scores and may also be compared with preoperative and postoperative scores. However, nasometer scores may also be influenced by articulation errors, particularly if the child uses compensatory articulation with nasal realizations instead of pressure consonants. Furthermore, upper respiratory infection or common cold might influence the scores making the test unreliable. Nasometry cannot diagnose the cause of velopharyngeal dysfunction and does not provide data for the velopharyngeal gap; nasal air emissions may also be the result of an oronasal fistula instead of VPI. With knowledge of speech characteristics, the speech pathologist can use nasometry to confirm the results of the perceptual assessment.

### Nasoendoscopy

**FIGURE 3:**
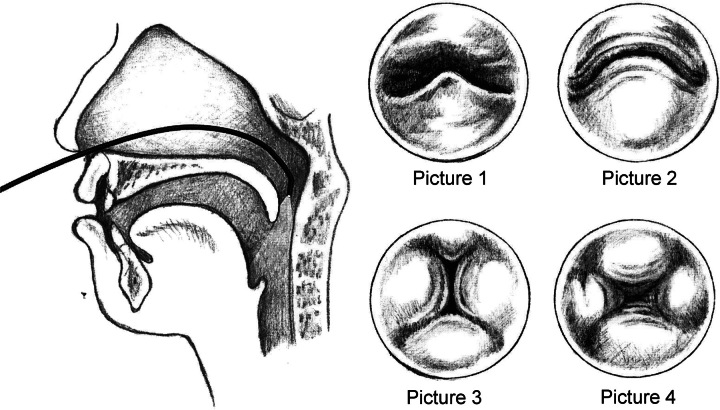
On the left side, a picture of nasoendoscopy method. On the right-side, bird’s-eye view by nasoendoscopy of the velopharyngeal sphincter: Picture 1 on rest, Picture 2 of a coronal closure pattern, Picture 3 of a sagittal closure pattern, and Picture 4 of a circular closure pattern. Original picture was presented in Avoin Hymy.^35^

Pigott and colleagues introduced nasopharyngeal endoscopy in 1969.^34^ As the technology has improved significantly and nasoendoscopy provides an optimal bird’s-eye view of closure of the velopharyngeal sphincter (Fig. 3), the position and function of the levator muscles, the length and quality of the soft palate, and the motion of the lateral pharyngeal walls and posterior pharynx.^36^ Nasoendoscopy provides important knowledge of the velopharyngeal sphincter’s closure pattern and helps to determine the optimal VPI surgery method.^37^ Furthermore, it can be useful to assess oronasal fistulae, submucous clefting, palatal scarring, tonsils, and adenoids. Nasoendoscopy should be done in High-Definition video recorded with sound on and performed whereas patients repeat standardized speech samples personalized to each patient’s abilities. A major limitation is challenge of compliance during examination of pediatric patients. Children aged over 4 years can cooperate enough to undergo nasoendoscopy without sedation. Nasoendoscopy might be easier for younger patients with light sedation. A study has proposed that nasoendoscopy should be performed intraoperatively under light sedation to achieve better compliance and patient comfort.^38^ However, the decision regarding surgical method is impossible to make in advance when performing the nasoendoscopy intraoperatively. Another limitation is that anatomical findings, such as the size of structures and extent of velopharyngeal closure, are impossible to quantitively measure, because the scope position affects the distance to different structures and spatial measurements, leading to subjectivity of interpretation of the anatomical findings.^39^ Furthermore, nasoendoscopy provides only one image plane (the axial), and the movements of pharyngeal walls can be difficult to interpret.^40,41^ Several studies have investigated the reliability of nasoendoscopy, most concluding it to be reliable,^42–44^ but some disagree.^40,45^ Nasoendoscopy has recently advanced to include 3-dimensional imaging using coronal, sagittal, and axial planes,^46^ but this approach is not yet widespread in clinical practice.

### Videofluoroscopy

**FIGURE 4:**
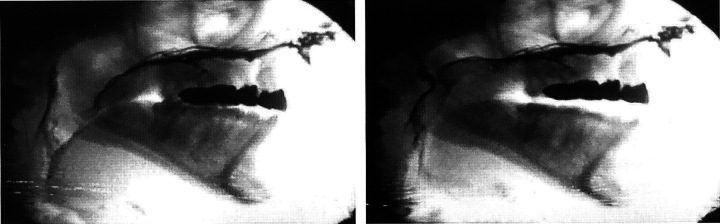
Lateral view by videofluoroscopy with barium given transnasally. Picture on the left side on rest and on the right side during speech; velopharyngeal sphincter does not reach to close during speech. Original picture was presented in Avoin Hymy.^35^

The procedure was first described by Skolnick in 1970.^47^ Videofluoroscopy involves imaging through multiple planes allowing measurements and should be videorecorded during speech with sound on. Multiple views, including lateral (sagittal), anteroposterior, and base or Towne, are often needed to obtain a clear visual of the velopharyngeal sphincter during a speech sample. In collaboration with a radiologist and speech pathologist, the procedure is performed during a standardized speech sample personalized to each patient’s abilities. High-density contrast material, barium, given transnasally during the examination allowing better visualization of the velopharyngeal sphincter (Fig. 4). Videofluoroscopy is usually performed in both lateral and anteroposterior views providing images of the posterior pharyngeal wall and Passavan ridge. Lateral view provides information regarding the extent of velar movement superiorly and posteriorly, and the contribution of the posterior wall. Frontal view assesses lateral pharyngeal wall movement. Basal view assesses the relationship between the velum and pharyngeal wall, the closure pattern of the velopharyngeal sphincter, and is analogous to the nasoendoscopic view. Videofluoroscopy provides quantitive information with real-size measurements by ratio of selected distance markings (de Stadler and Hersh, 2015), but may oversimplify the velopharyngeal sphincter, making patterns of closure difficult to assess,^48^ and creating an overestimation of velopharyngeal closing.^49^ Another limitation is the fact that the procedure requires exposure to radiation, although a limited amount. It also requires co-operation from the patient. Recent reports describe that using appropriate methods for performing speech videofluoroscopy produces only < 9 mSv (millisievert) dosages of radiation.^50,51^ Compared with nasoendoscopy, videofluoroscopy is often better tolerated,^52^ and is usually more usable in younger children, although application of transnasal barium can raise resistance in young children.^49^ Some centers use only the lateral view to increase the ability to conduct imaging of small children and decrease the amount of radiation.^53^

### Magnetic Resonance Imaging

Magnetic resonance imaging has been promoted for the evaluation of the velopharynx, providing high-quality, reproducible, static, dynamic, 2-dimensional and 3-dimensional imaging. Unlike videofluoroscopy, MRI does not require radiation exposure. Some studies have described synchronizing audio and video samples during speech without delay, making real-time dynamic visualization in combination with speech assessment possible.^54,55^ However, MRI sometimes requires sedation in younger children to reduce motion artefacts,^56^ and still remains cost prohibitive.^49,54^ Magnetic resonance imaging may offer a radiation-free, better tolerated and more precise diagnostic tool of velopharyngeal sphincter function in the future.^57^ Perry and her colleagues have done comprehensive research on MRI in velopharyngeal anatomy evaluation, establishing the role of MRI in 3-dimensional imaging of velopharyngeal anatomy,^58,59^ dynamic evaluation of velopharyngeal sphincter during speech,^56,60–62^ and often with no use of sedation.^63,64^ Furthermore, the use is still limited and under examination, but ongoing studies will show if MRI will have the potential to be the new standard diagnostic tool of velopharyngeal sphincter.

## MANAGEMENT OF VPI

The definitive treatment of VPI in patients with a history of cleft palate is VPI surgery due to the anatomical cause of the condition.^2,65^ A prosthetic device can be used if surgical treatment is not an option or temporarily. Speech therapy should be reserved for compensatory articulation.

### Conservative Treatment

Speech therapy is the mainstay treatment for velopharyngeal mislearning and compensatory articulation,^2,66^ but it cannot improve hypernasality, nasal emissions, or weak pressure consonants.^67^ Although speech therapy can address compensatory articulation and not VPI, a common misconception about the role of speech therapy in the management of “cleft palate speech” still exists.^32^ Traditionally children with CP and VPI might have been referred to speech therapy before deciding on VPI surgery even when the cause is anatomical. Ruscello has addressed issue of speech therapy and especially oral motor exercises (blowing, sucking, etc.) as primary rehabilitation without VPI surgery in treatment of VPI and concluded that such treatment is ineffective, benefitting only few patients.^68,69^ However, it is essential that speech therapy continues after VPI surgery when compensatory articulation and velopharyngeal mislearning exists.

Prosthetic devices can be used as temporary treatment or if surgical treatment is not an option. These devices are custom-made. An obturator is a removable maxillary prosthesis which restores the defects of the soft palate and fills the gap between the soft palate and posterior pharyngeal wall when the palate is short in case of VPI.^70^ A palatal lift elevates the soft palate when it is unable to contact the posterior pharyngeal wall due to neurogenic dysfunction in case of velopharyngeal incompetence.^70^

### Surgical Treatment

Surgical management is usually first line of treatment for VPI. The aim of surgical treatment is to create a functional seal in the velopharyngeal sphincter during speech, while avoiding airway obstruction. Multiple methods have been described to treat VPI. Methods for VPI surgery are either static or functional in design: static methods aim to compensate the poor function by narrowing the velopharyngeal sphincter and functional methods aim to correct muscle function.

### Static Methods

Static methods are traditionally used in VPI treatment after primary palatoplasty and they aim to obstruct the velopharyngeal sphincter to achieve velopharyngeal closure. Pharyngeal flap is probably still the most used method followed by sphincter pharyngoplasty: a systematic review of surgeries for velopharyngeal dysfunction showed that 64% of studied patients underwent pharyngeal flap and 24% sphincter pharyngoplasty.^30^

#### Pharyngeal Flap

Passavant first described pharyngeal flap in 1865^71^ and Hogan popularized the method in the 1970s.^72^ Pharyngeal flap is one of the oldest and still the most commonly used surgical methods for treating VPI.^30^ A musculomucosal bridge is created from the posterior pharyngeal wall to the soft palate to divide the velopharyngeal sphincter into 2 smaller ports and decrease airflow during speech.^72^ The flap can be either superiorly or inferiorly based and many other modifications exist including not splitting the palate in the midline,^73^ not lining the flap with nasal mucosa from the soft palate,^73^ and performing a through dissection of the soft palate for flap insert.^74^ A recent systematic review of surgical management of velopharyngeal dysfunction shows 76% of patients achieving normal resonance with 6% revision rate after pharyngeal flap^30^ and no recent studies have shown one posterior pharyngeal flap modification to be superior to another. Perioperative complication rate of pharyngeal flap is quite low around 5%^75^ but long-term complications are common. The most common long-term complication of pharyngeal flap is obstructive sleep apnea (OSA), seen in as many as 20% to 90% of patients postoperatively.^76–80^ OSA after pharyngeal flap leads to reoperations, however flap takedown does not necessary lead to deleterious speech outcome.^81^

#### Sphincter Pharyngoplasty

Sphincter pharyngoplasty was introduced by Hynes in the 1950s^82^ and includes elevation of bilateral thick vertically oriented posterior tonsillar pillar flaps, including the palatopharyngeus muscle, and transposing them 90 degrees medially to a horizontal position, against the posterior pharyngeal wall. Modifications of the original procedure include augmentation of the muscle sphincter created by the posterior tonsillar pillar flaps with the longus capitis muscle, cerclage of the sphincter pharyngoplasty using polypropylene sutures, and placing the pharyngoplasty higher in the pharynx.^83–85^ On the basis of a recent comprehensive review of 2033 nonsyndromic children with CP undergoing sphincter pharyngoplasty, the success rate of sphincter pharyngoplasty is 78% and revision rate 18%.^86^ However, as sphincter pharyngoplasty also aims for obstruction of the velopharyngeal sphincter, postoperative OSA is an issue. In the same review by Grover and colleagues, 353 children underwent evaluation of OSA and overall postoperative rate of OSA was 18%.^86^

#### Posterior Wall Augmentation

Posterior wall augmentation involves placing augmentation material within the posterior pharyngeal wall narrowing the velopharyngeal gap. Many types of material have been used, including autologous fat, acellular dermis,^87^ autologous cartilage,^88^ calcium hydroxylapatite,^89^ and hyaluronic acid.^90^ Posterior wall augmentation is usually used in case of mild VPI and small velopharyngeal gaps.^91^ A systematic review by de Blacam and colleagues reported only 51% of patients achieving normal resonance with 19% revision rate after posterior wall augmentation.^30^ Although a review of autologous fat grafting by Bishop and colleagues revealed only one published report of a serious complication,^92^ the safety and long-term results of the procedure remain unproven as the method is relatively new. As presented in the review by Bishop and colleagues, Teixeira and colleagues reported a case of obstructive sleep apnea after fatty hypertrophia.^93^ An unpublished British report also described a middle cerebral artery infarct after a fat embolism in a young patient after autologous fat injection for VPI treatment.^94^

### Re-palatoplasty Methods

Palatal re-repair methods include palatal re-repair with intravelar veloplasty^95^ and double-opposing Z-plasty,^96^ and they aim for anatomical muscle repair and pharyngeal ring reconstruction by correcting the abnormal position of the levator veli palatini, suturing the muscle ring, and removal of scar tissue. Both have been advocated for the treatment of VPI in patients who initially underwent a straight-line palatoplasty and/or demonstrate anterior insertion of the levator veli palatini. Despite the benefits of these functional methods, in a recent systematic review of VPI treatments, only 8% of patients received palatoplasty (in which they included palatal re-repair with intravelar veloplasty, double-opposing Z-plasty, pushback, buccinator flaps, and fat injection to the palate).^30^ A review by Kurnik and colleagues of the efficiency of re-repair procedures, including palatal re-repair with or without mucosal lengthening and double-opposing Z-plasty, reported postoperative overall incidence of achieving no consistent hypernasality in only 61% of patients but noted substantial heterogeneity across studies for all outcomes.^97^ De Blacam and colleagues review showed improved resonance in 73% after palatoplasty method for VPI, but completely normal resonance in only 58%^30^; however, these numbers include not only palatal re-repair with intravelar veloplasty and double-opposing Z-plasty, but also pushback, buccinator flaps, and fat injections to the soft palate. The advantage of palatal re-repair methods is the lesser risk for OSA as these methods do not aim to obstruct the velopharyngeal sphincter. The review by Kurnik and colleagues showed OSA in 28% after re-repair procedures compared with 86% after pharyngeal flap with the risk being substantially lower after re-repair than pharyngeal flap.^97^

#### Palatal Re-repair with Intravelar Veloplasty

Dellon and Edgerton first described palatal re-repair with intravelar veloplasty, for VPI treatment in 1969,^98^ and Sommerlad later popularized the method.^95,99^ Sommerlad’s principles for muscle repair can be used in primary palatoplasty^100^ and palatal re-repair for VPI.^95,99^ The method involves opening the velum trough a midline approach with careful and radical dissection of the abnormally inserted palatal muscles and Sommerlad also advices the use of an operating microscope.^95^ Palatal re-repair by Sommerlad has been shown to be effective (meaning postoperative normal resonance and nasal airflow or mild and inconsistent hypernasality, nasal emission, or turbulence) in 82% of cases.^95^

#### Double-opposing Z-plasty

Furlow first described palatoplasty using 2 opposing Z-plasties of the soft palate, redirection of the palatal muscles to produce an overlapping sling, and dissection of the palatal muscles from only one of the 2 mucosal covers.^101^ The method was later introduced for secondary surgery to treat VPI.^102^ The geometric design of the double-opposing Z-plasty achieves 2 opposite Z-plasties of both the nasal and oral mucosa and a transverse orientation of the levator veli palatini muscles, displacing them anatomically overlapping each other. This approach achieves lengthening of the velum, reorientation of the levator veli palatini muscles, and narrowing of the velopharyngeal sphincter. Many modifications have been introduced to the original method, including: Langenbeck-type relaxing incisions to accomplish a tension-free closure,^102,103^ leaving the nasal layer intact^96^; and separating the muscle bundle from the oral and nasal mucosa, and retro positioning the levator ring.^96^

#### Buccal Flap

Buccal flaps have been used for several indications in craniofacial surgery, including closure of palatal fistulas and primary and secondary cleft palate repair. As the method lengthens the palate, it has been introduced to VPI surgery method. Mann popularized the method in both primary and secondary palatal surgery in recent years.^104–106^ The method can be used uni-^107^ and bilaterally,^105^ and it can also be combined with Furlow palatoplasty.^106^ The use of buccal flaps lengthens the palate^108^ and often allows tension-free closure; however, it rarely aims for anatomical palatal muscle reconstruction and yet no long-term results have been studied.^109^

## SUMMARY

Velopharyngeal insufficiency is a common complication after primary palatoplasty. Instrumental and imaging methods including nasometry, nasoendoscopy, and videofluoroscopy are widely used for diagnosing VPI; future studies will show if MRI will be the future standard imaging method for velopharyngeal anatomy. Careful monitoring of VPF by a speech pathologist remains paramount for early identification of VPI, but the perceptual assessment should follow a standardized protocol, which is still rarely used. The greatest methodology problem in CLP studies has been the use of highly variable speech samples making comparison of published results impossible. It is hoped that ongoing international collaborative efforts to standardize procedures for collection and analysis of perceptual data will help this issue. On the basis of the current literature, speech therapy is the mainstay treatment for velopharyngeal mislearning and compensatory articulation, but it cannot improve hypernasality, nasal emissions, or weak pressure consonants, and surgery is the definitive treatment for VPI. Although many surgical methods are available, there is no conclusive data to guide procedure choice.

## Supplementary Material

SUPPLEMENTARY MATERIAL
