# Supplementary material for: Diagnosing and Managing Velopharyngeal Insufficiency in Patients With Cleft Palate After Primary Palatoplasty
Source: J Craniofac Surg. 2023 Nov 13;36(3):1008–16. doi: 10.1097/SCS.0000000000009822 (PMC12020403; doi:10.1097/SCS.0000000000009822)
Supplement: SUPPLEMENTARY MATERIAL [file scs-36-1008-s001.docx]

**Tables Supplemental**

**Supplemental Table 1** Instrumental evaluation methods; evaluation method, assessment of VPF, technique, advantages and disadvantages.

| Evaluation method | Assessment of VPF | Technique | Advantages and disadvantages |
| --- | --- | --- | --- |
| Nasoendoscopy  (Havstam et al., 2005; Berkowitz, 2013; Shprintzen, 2013) | A direct visualization of the vocal tract and the velopharyngeal structures, their movement and the size, location, and consistency of the velopharyngeal gap | A flexible endoscope inserted from one side of the patient’s nasal cavity to visualize the velopharyngeal area or the vocal tract | -direct anatomic view  -ability to see the whole vocal tract  -no time limit, no radiation  -invasive, slightly uncomfortable |
| Videofluoroscopy  (Havstam et al., 2005; Berkowitz, 2013; Shprintzen, 2013) | A direct radiographic method providing information about the width, depth and height of the velopharyngeal valving | Barium inserted in each nostril covering the lateral and posterior parts of the velopharyngeal area | -vocal tract and articulators seen simultaneously  -painless  -slightly invasive and uncomfortable  -exposure to radiation |
| Nasometer  (Dalston et al., 1991; Kuehn and Moller, 2000; Berkowitz, 2013) | Acoustic tool to measure a nasalance score representing the ratio of the oral and nasal acoustic energy | A sound separator placed on the upper lip with microphones in front of the nose and the mouth | -easy to use  -inexpensive  -noninvasive, painless  -no direct view to the velopharyngeal valving |

**Supplemental Table 2** Standardized methods for perceptual evaluation of cleft speech.

| The Scandcleft methodology | Designed to enable cross-linguistic comparison of speech outcomes after different primary surgeries (20,105,106). |
| --- | --- |
| CAPS-A | The Cleft Audit Protocol for Speech–Augmented used in U.K and Ireland (11,14,107), USA (108) and in Belgium (109) for intercenter audit studies and for reporting speech outcomes after surgical treatment. |
| SVANTE | The Swedish Articulation and Nasality Test (21) used widely in the Nordic countries. Inspired by the Scandcleft project and designed to enable crosslinguistic evaluation. |
| TOPS | Designed to enable cross-linguistic comparison of speech outcomes after different timing of primary surgeries, based on the Scandcleft methodology (Shaw et al. 2019, Willadsen et al. 2023). |

**Supplemental Table 3** Differences between the standardized methods for perceptual evaluation of cleft speech.

| Method | Language | Speech material | Number of words | Ratings | Overall VPF Scale |
| --- | --- | --- | --- | --- | --- |
| Scandcleft methodology | Restricted (Danish, English, Finnish, Norwegian, Swedish) | single words, continuous speech | 30-33 | Consonants (active characteristics: anterior oral, posterior oral, posterior non‑oral/passive characteristics: nasal air leakage, weakness), hypernasality, hyponasality, velopharyngeal closure, velopharyngeal composite score (VPC-Sum) | VPC-Sum (0-6:   - 1. competent,   2-3 marginally incompetent,  4-6 incompetent)  VPC-Rate (0-6:  0-2 competent,  2-3 marginally incompetent,  4-6 incompetent) |
| CAPS-A | Language specific (English, Dutch) | rote speech (counting etc.), sentence repetition, continuous speech | - | Intelligibility, voice, hypernasality/ hyponasality, nasal emissions/nasal turbulence, grimance, anterior/posterior/non-oral/passive CSCs | Not originally  Later validated VPC-Sum CAPS-A (0-4:   - 1. sufficient,   2 borderline deficit,  3-4 insufficient) |
| SVANTE | Language specific (Swedish) | single words, sentences, continuous speech | 59 | Articulation (oral consonants, errorrs in front of VPF, errors behind of the VPF), nasality (hypernasality, hyponasality, audible nasal air leakage, perceived reduced pressure on consonants), overall measures (velopharyngeal function, intelligibility), phonology number (established phonemes, consonant processes) | VPF (0-2:  0 Competent/sufficient,  1 marginally incompetent/insufficient,  2 incompetent/insufficient)  VPC-Rate (Scandcleft methodology) |
| TOPS | Restricted (Danish, English, Norwegian, Portuguese, Swedish) | single words, continuous speech | 30-36 | Consonants (active characteristics: anterior oral, posterior oral, posterior nonoral/passive characteristics: nasal air leakage, weakness), hypernasality, hyponasality, velopharyngeal closure, velopharyngeal composite score (VPC-Sum), VPC-Rate | VPC Sum (Scandcleft methodology),  VPC-rate (Scandcleft methodology) |
